# Direct Medical Costs of Advanced Breast Cancer Treatment: A Real-World Study in the Southeast of The Netherlands

**DOI:** 10.1016/j.jval.2020.12.007

**Published:** 2021-05

**Authors:** Paul Peter Schneider, Bram L. Ramaekers, Xavier Pouwels, Sandra Geurts, Khava Ibragimova, Maaike de Boer, Birgit Vriens, Yes van de Wouw, Marien den Boer, Manon Pepels, Vivianne Tjan-Heijnen, Manuela Joore

**Affiliations:** 1 Department of Clinical Epidemiology and Medical Technology Assessment, Maastricht University Medical Centre+, Maastricht, The Netherlands; 2 School of Health and Related Research, University of Sheffield, Sheffield, UK; 3 Department of Medical Oncology, GROW – School of Oncology and Developmental Biology, Maastricht University Medical Centre+, Maastricht, The Netherlands; 4 Catharina Hospital, Eindhoven, The Netherlands; 5 VieCuri Medical Center, Venlo, The Netherlands; 6 Laurentius Hospital Roermond, The Netherlands; 7 Elkerliek Hospital, Helmond, The Netherlands

**Keywords:** breast neoplasms, healthcare utilization, healthcare costs, real-world data, The Netherlands

## Abstract

**Objectives:**

Policy makers increasingly seek to complement data from clinical trials with information from routine care. This study aims to provide a detailed account of the hospital resource use and associated costs of patients with advanced breast cancer in The Netherlands.

**Methods:**

Data from 597 patients with advanced breast cancer, diagnosed between 2010 and 2014, were retrieved from the Southeast Netherlands Advanced Breast Cancer Registry. Database lock for this study was in October 2017. We report the observed hospital costs for different resource categories and the lifetime costs per patient, adjusted for censoring using Lin’s method. The relationship between patients’ characteristics and costs was studied using multivariable regression.

**Results:**

The average (SE) lifetime hospital costs of patients with advanced breast cancer were €52 709 (405). Costs differed considerably between patient subgroups, ranging from €29 803 for patients with a triple-negative subtype to €92 272 for patients with hormone receptor positive and human epidermal growth factor receptor 2 positive cancer. Apart from the cancer subtype, several other factors, including age and survival time, were independently associated with patient lifetime costs. Overall, a large share of costs was attributed to systemic therapies (56%), predominantly to a few expensive agents, such as trastuzumab (15%), everolimus (10%), and bevacizumab (9%), as well as to inpatient hospital days (20%).

**Conclusions:**

This real-world study shows the high degree of variability in hospital resource use and associated costs in advanced breast cancer care. The presented resource use and costs data provide researchers and policy makers with key figures for economic evaluations and budget impact analyses.

## Highlights

• Accurate information on the costs of medical treatment is required not only from clinical trials, but also from the real world, to inform health policy decision making, especially in high-cost areas such as cancer therapy.
• This study provides a detailed analysis of the real-world medical costs of patients with advanced breast cancer in The Netherlands.
• Considerable variation in costs and resource use were observed between and within patient subgroups.
• Reported cost estimates can be used to improve health economic evaluations of medical interventions.

## Introduction

Breast cancer is the most common type of cancer among women and a leading cause of death worldwide*.*^1^ Despite significant improvements in the early diagnosis and treatment of breast cancer, about 5% of the patients present with advanced (ie, metastatic) disease at the time of diagnosis, and a further 20% of patients will experience progression to advanced disease later in life.^2^ Even though some patients live with advanced breast cancer (ABC) for many years, the disease is considered incurable, and the main objective of care is to prolong survival and sustain quality of life. Due to its high prevalence and high individual treatment costs, the economic burden of ABC in The Netherlands, as well as in many other countries, is substantial.^3^, ^4^, ^5^, ^6^

Over the last 2 decades, several new drugs for the treatment of ABC have become available. Many are highly expensive, posing a substantial economic burden on society and raising questions regarding their value for money.^6^^,^^7^ Health economic evaluations are essential to inform reimbursement decisions for these novel agents.

Although randomized controlled trials (RCT) are considered the gold standard for supporting preapproval efficacy, their value for health economic evaluations and real-world effectiveness is limited. Resource utilization is rarely being considered in RCTs, and even if relevant data are collected, treatments are often tested under artificial circumstances. In routine care, patients often have more comorbidities, lower compliance, are older, and/or treatment patterns are different from patients included in RCTs.^8^ Furthermore, a significant proportion of the resource utilization may fall beyond the trial horizon. In health economic evaluations, healthcare costs frequently have to be based on expert opinions and/or taken from different patient populations and other settings. Therefore, policy makers increasingly seek to complement data collected in RCTs with data from routine care.^9^

Under real-world conditions, ABC care is complex: patients are highly heterogeneous and treatment choices and pathways are individualized and depend on patient characteristics, treatment responses, and preferences.^10^ Therefore, aggregate cost estimates computed from administrative data are of limited value, and decision makers should aim to take into account all the relevant factors to prescribe optimal policies. Up until today, however, little is known about the real-world hospital resource use and the associated costs in patients with ABC.^11^^,^^12^ The complexities of ABC care require the information to be comprehensive, granular, and contextual, to guide decision making in The Netherlands and in other countries.

This study aims to investigate the real-world resource use and costs of ABC in The Netherlands from the hospital perspective. First, we assessed patients’ resource utilization and the associated costs; second, we estimated the average lifetime costs of patients with ABC; and, finally, we investigated which factors contribute to the heterogeneity of costs between patients.

## Methods

### Patient and Data Collection

This study used patients included from the Southeast Netherlands Advanced Breast Cancer (SONABRE) Registry.^13^ The ongoing registry was initiated in 2010 and aims to include all patients with de novo or recurrent ABC, diagnosed at age 18 years or older, from 12 participating hospitals in the southeast of The Netherlands*.* For this study, we used data from the 5 hospitals, in which information on resource use was collected from 2010 through 2017. These hospitals were selected with the intention to obtain a representative mixture of different hospital types and sizes, and they account for approximately 7% of the hospitals in The Netherlands. Patients were included if they were diagnosed with ABC between 2010 and 2014. No exclusion criteria were employed. Data lock was October 23, 2017.

Patients were identified prospectively, and clinical data were, retrospectively, retrieved manually from electronic medical records by trained registration clerks and entered into a registry database. Collected patient and disease characteristics included age, survival time, comorbidities, and tumor characteristics (initial hormone receptor [HR] and human epidermal growth factor receptor 2 [HER2] status). Moreover, we retrieved data on hospital resource use for the following categories: medicines/systemic therapies (chemotherapy, endocrine therapy, targeted therapy, bone-modifying agents/bisphosphonates, and transfusions), consultations/hospitalizations, radiotherapy, and diagnostic and surgical procedures.

### Handling of Incomplete and Missing Data

Because our study was based on routinely collected data, missingness was unavoidable. This problem was mostly limited to medications, for which the administered dosage was missing, while the number of administrations was reported. To avoid the creation of implausible observations, we used hot deck imputation to replace missing values with observed values from another patient, matched by the resource they used.^14^ For hormonal therapies and bone-modifying agents/bisphosphonates, consumed resource units were not recorded in the registry but were computed based on treatment durations and respective standard doses.^15^ When a patient’s treatment duration was not reported, resource consumption was imputed using the agent-specific average duration. If the HER2 (n = 58) and/or HR (n = 3) receptor status is not tested, patients are being treated as if they had negative receptor status. In our study, such patients were classified accordingly as triple negative (TN).

### Resource Use and Associated Costs

For all patients, we assessed the hospital resource use and associated costs from the date of diagnosis (ie, date of pathological conformation, or else date of imaging) until date of death or censoring. Costs associated with resource consumption were derived by multiplying the units of resource consumption with the respective cost prices. Relevant unit costs were taken from Dutch costing guidelines, medicijnkosten.nl, and, if otherwise unavailable, from individual studies or the financial department of Maastricht University Medical Centre (see Appendix Table 1 in Supplemental Materials found at https://doi.org/10.1016/j.jval.2020.12.007).^16^^,^^17^ All costs are expressed in 2017 Euro. If necessary, costs were inflated to the price level of this year, using the consumer price index.^18^

For each type of resource, we assessed the average consumption and the average costs, conditional and unconditional on resource use, as well as the share on total costs. Since cost estimates are provided for use in health economic decision modeling, we only report averages and their bootstrapped 95% confidence intervals, which are commonly used in models, instead of, for example, the median, as a more stable measure of central tendency. For drugs, consumption is expressed in terms of the most commonly administered dosage. The hospital costs associated with administering drugs intravenously are reported separately, as they have been previously reported to account for a considerable share of total costs.^19^

### Lifetime Costs

We calculated the average costs per patient, as well as the costs per patient month. Since survival time and lifetime costs tend to be correlated, the observed costs per patient are not an unbiased estimate of lifetime costs, in the presence of censoring. Including patients based on the date of diagnosis enabled us to apply adjustment methods to extrapolate the observed costs for any patient who was censored over a lifetime horizon. To adjust the costs for censoring, we used the approach described by Lin et al^20^: the observation period was split into intervals, based on dates of death and censoring. For each interval, we computed the mean and weighted it by the Kaplan-Meier estimator (ie, the probability that patients survive until the beginning of the interval). We then summed up the weighted means to derive an estimate of mean lifetime costs of ABC. To compute standard errors around means, we used bootstrapping with 1000 iterations. When appropriate, we refer to the observed costs as being unadjusted.

### Heterogeneity of Costs Between Patients

We further investigated the heterogeneity in costs, that is, the share of the variability that can be explained by patient and tumor characteristics. The following factors, identified from the literature and/or clinical expertise, were taken into consideration^12^: survival time, age, year of diagnosis, interval between primary diagnosis of breast cancer and diagnosis of metastatic disease, initial HER2 and HR status, death (in versus outside of a hospital), systemic therapy (was any systemic therapy initiated?), and locoregional aggressive treatment (defined as breast surgery or radiotherapy with 15 or more fractions within the first year after diagnosis of metastatic disease).

We investigated the association between these variables and 2 outcomes: observed total costs per patient and costs per patient month. Although the former was the main focus of this study, survival time was assumed to explain a large proportion of the variability in total costs, so that inference about other variables may be limited. For both outcomes variables, we fitted generalized linear models, with a gamma distribution and a log link. This method, which is frequently used to model cost data, was chosen to evade the shortcomings of ordinary least squares, specifically with regard to right-skewed data, heteroscedasticity, and the strictly non-negative values in cost data.^21^ An ordinary least squares model may, for example, predict negative expenditure (ie, gains) for short periods of survival time. We used backward elimination to successively remove predictors and find the model with the lowest Bayesian information criterion.^22^ As a goodness-of-fit measure, we report the McFadden’s pseudo *R*^2^ value. A sensitivity analysis was conducted to investigate whether effect estimates for the subsample of deceased patients differed compared to the full cohort (including deceased and censored patients). Alternative generalized linear model specifications (Gaussian distribution with a log link and Gaussian distribution with an identity link) were also tested to validate our model choice.

### Ethical Approval

The Medical Research Ethics Committee of Maastricht University Medical Centre+ reviewed and approved the SONABRE Registry. The need for informed consent was waived because of the observational nature of this study.

## Results

### Patient Population

After the application of the inclusion criteria, 597 patients from the SONABRE registry were included in the study. The number of patients per year of ABC diagnosis varied only a little between 2010 and 2014, with the minimum being in 2014 (n = 103; 17%) and the maximum being in 2011 (n = 128; 21%). Overall, 436 patients died and 161 were censored. The median survival time was 24.5 months (95% CI 22.7-27.5). The median follow-up time (ie, time until censoring) was 55.2 months (51.7-58.7). A large majority of the patients in our cohort had a HR+/HER2- receptor status at the time of the initial diagnosis (n = 417; 70%), followed by TN (n = 69; 12%); HR+/HER2+ (n = 65; 11%), and HR-/HER2+ (n = 46; 8%). A total of 199 patients (33.3%) had any of the following comorbidities: metabolic disease (n = 90; 15%), cerebral disease (n = 32; 5%), cardiovascular disease (n = 63; 11%), other malignancy (n = 62; 10%), or pulmonary disease (n = 43; 7%). Further patient characteristics are provided in Table 1.

**Table 1:** Patient characteristics.

Variable	Value
Sample size, n	597
Age (years), Median (IQR)	64 (55-74)
Metastasis-free interval	
<3 months, n (%)	136 (23)
3-24 months, n (%)	89 (15)
>24 months, n (%)	372 (62)
Survival (months), Median (95% CI)	24.5 (22.7-27.5)
Censored, n (%)	161 (27)
Deceased, n (%)	436 (73)
Death in hospital, n (%)	113 (19)
Death outside hospital, n (%)	323 (54)
Treatments	
Any systemic therapy, n (%)	532 (89)
Locoregional aggressive treatment- n (%)	44 ( 7)
No systemic therapy, n (%)	65 (11)
Year of ABC diagnosis	
2010, n (%)	107 (18)
2011, n (%)	128 (21)
2012, n (%)	125 (21)
2013, n (%)	134 (22)
2014, n (%)	103 (17)
Initial HR/HE=R2 receptor status	
HR+/HER2-, n (%)	417 (70)
HR+/HER2+, n (%)	65 (11)
HR-/HER2+, n (%)	46 ( 8)
TN, n (%)	69 (12)
Comorbidities	
Metabolic disease, n (%)	90 (15)
Cerebral disease, n (%)	32 (5)
Cardiovascular disease, n (%)	63 (11)
Other malignancy, n (%)	62 (10)
Pulmonary disease, n (%)	43 (7)

HER2 indicates human epidermal growth factor receptor 2; HR, hormone receptor; IQR, interquartile range; TN, triple-negative (HR-/HER2-).

### Resource Use and Associated Costs

Table 2 provides an overview of the hospital resource use and the associated costs in our cohort as observed during the study period. Shown are the number of patients who used a particular resource and the associated share in total costs, as well as the average units of resource consumption and the average costs per patient, conditional on resource use (ie, the average for those patients, who used the resource). In addition to the figures for aggregate resource categories, data on individual resource items are reported if the respective share in total costs was at least 1%. Total costs refer to the sum of costs of all included patients over the entire observational period. For around 1.2% of the consumed resources, the number of consumed units was not reported and had to be imputed. HR/HER2 subtype-specific resource use and cost figures are provided in Appendix Tables 2-5 in Supplemental Materials found at https://doi.org/10.1016/j.jval.2020.12.007.

**Table 2:** Resource use and associated costs in €.

Resource	Observed total costs	% of total costs	% of costs within category	Uncond. average costs per patient	Used by patients n (%)	Cond. average∗ units consumed (standard dose†)	Cond. average∗ costs per patient (95% CI)
Targeted therapy (any)	9 720 764	36.6%		16 283	256 (43%)		37 972 (33 691-42 084)
Trastuzumab	3 987 004	15.0%	41.0%	6678	77 (13%)	21.49 (600 mg)	51 779 (42 394-61 628)
Everolimus	2 598 371	9.8%	26.7%	4352	119 (20%)	149.75 (10 mg)	21 835 (18 457-25 368)
Bevacizumab	2 516 255	9.5%	25.9%	4215	80 (13%)	15.57 (600 mg)	31 453 (27 021-36 958)
T-DM1	362 859	1.4%	3.7%	608	11 (2%)	7.69 (230 mg)	32 987 (20 772-46 645)
Chemotherapy (any)	2 430 719	9.2%		4072	307 (51%)		7918 (7120-8738)
Doxorubicin	890 660	3.4%	36.6%	1492	91 (15%)	11.11 (40 mg)	9787 (8856-10 754)
Paclitaxel	699 671	2.6%	28.8%	1172	131 (22%)	15.34 (140 mg)	5341 (4774-5962)
Docetaxel	285 374	1.1%	11.7%	478	68 (11%)	4.32 (193 mg)	4197 (3714-4694)
Hormonal therapy (any)	912 051	3.4%		1528	415 (70%)		2198 (1830-2606)
Fulvestrant	869 001	3.3%	95.3%	1456	170 (28%)	7.87 (500 mg)	5112 (4343-5946)
Bisphosphonates (any)	1 613 553	6.1%		2703	316 (53%)		5106 (4708-5503)
Pamidronic acid	563 132	2.1%	34.9%	943	151 (25%)	20.50 (90 mg)	3729 (3223-4237)
Denosumab	553 899	2.1%	34.3%	928	75 (13%)	17.19 (120 mg)	7385 (6489-8293)
Clodronic acid	432 082	1.6%	26.8%	724	122 (20%)	545.65 (1600 mg)	3542 (3196-3905)
Transfusions	152 105	0.6%		255	122 (20%)		1247 (1039-1477)
Systemic therapy administration	164 060	0.6%		275	154 (26%)		1065 (938-1198)
Consultations (any)	7 882 104	29.7%		13 203	591 (99%)		13 337 (12 446-14 206)
Inpatient day	5 199 722	19.6%	66.0%	8710	458 (77%)	17.55	11 353 (10 510-12 108)
Outpatient visit	2 035 273	7.7%	25.8%	3409	582 (97%)	26.05	3497 (3315-3684)
ICU inpatient day	276 655	1.0%	3.5%	463	23 (4%)	9.97	12 028 (7525-17 700)
Emergency dep.	274 466	1.0%	3.5%	460	428 (72%)	2.43	641 (603-681)
Diagnostics (any)	2 362 807	8.9%		3958	589 (99%)		4012 (3806-4228)
CT thorax-abdomen	742 995	2.8%	31.4%	1245	452 (76%)	4.35	1644 (1528-1758)
CA 15.3 test	389 560	1.5%	16.5%	653	540 (90%)	13.95	721 (680-765)
Radiotherapy	890 405	3.4%		1491	307 (51%)		2900 (2523-3292)
Surgery (any)	417 049	1.6%		699	199 (33%)		2096 (1755-2430)

Individual resource costs are only shown for resources that accounted for at least 1% of total costs.

CI indicates confidence interval; ICU, intensive care unit.

∗ The conditional average per patient indicates the average resource use or costs conditional on having used the particular resource. Patients who were admitted at least once, for example, had an average of 17.55 inpatient days.

† For drugs, the average use per patient represents the average number of times the reported standard dose was administered, conditional on receiving the drug at least once.

A few specific points about the results shown in Table 2 are worth highlighting: First, the majority of costs (56%) of ABC treatment were related to systemic therapies. The main drivers of these costs were targeted therapies (37%) and bone-modifying agents/bisphosphonates (6%), whereby the individual agents trastuzumab (15%), everolimus (10%), bevacizumab (9%), and pamidronic acid (2%) accounted for the highest shares in costs. Second, of the 597 patients included in the study, a vast majority (89%) received 1 or multiple systemic therapies. Third, most patients had frequent contact with healthcare providers, and utilization of these services accounted for 30% of total costs. On average, outpatient clinics were visited about 26 times per patient, and 458 (77%) patients were admitted to a hospital at least once; admission was, on average, 18 inpatient days. Finally, many patients underwent comprehensive diagnostic testing and had multiple radiographic examinations (eg, CT scans, MRIs, x-rays), which contributed to the share of 9% on total costs.

### Lifetime Costs

Table 3 provides the mean costs per patient with ABC, both unadjusted (ie, as observed) and adjusted for censoring of patient time and costs. The unadjusted mean (95% CI) total costs per patient were €44 277 (41 949-44 277). The distribution of costs per patient had a wide range (min = €113; max = €298 769) and was heavily skewed (skewness = 1.92; kurtosis = 4.50), with a standard deviation of €44 943. Patients who died had considerably lower costs (€39 686 (95% CI 37 722-41 563)) than patients who were censored (€57 434 (95% CI 51 624-68 920)). After adjusting for censoring using Lin’s method, the mean (95% CI) lifetime costs per patient with ABC were estimated to be €52 709 (48 825-56 584). Patients with HR+/HER2+ ABC incurred the highest lifetime costs of €92 272 (69 447-105 947), followed by HR-/HER2+ with costs of €69 079 (58 375-82 295) and HR+/HER2- with costs of €47 495 (44 730-49 353). Patients with triple-negative ABC had the lowest lifetime costs of €29 803 (22 853-36 603). Differences between the (unadjusted) subgroup-specific mean costs per patient or patient month and the lifetime cost estimates were driven mainly by differences in survival time.^13^

**Table 3:** Costs of patients with advanced breast cancer – mean (95% confidence interval) in €.

	All patients	HR+/HER2-	HR+/HER2+	HR-/HER2+	TN
Monthly per patient costs (unadjusted)	1621 (1524-1725)	1275 (1190-1375)	2656 (2335-2986)	3076 (2499-3632)	2925 (2525-3371)
Lifetime per patient costs (unadjusted)	44 277 (41 949-46 606)	38 775 (36 008-40 193)	81 591 (75 342-96 585)	60 729 (50 392-71 699)	31 927 (29 573-38 317)
Lifetime per patient costs (adjusted for censoring)	52 709 (48 825-56 584)	47 495 (44 730-49 353)	92 272 (69 447-105 947)	69 079 (58 375-82 295)	29 803 (22 854-36 603)
n	597	416	65	45	71

HER2 indicates human epidermal growth factor receptor 2; HR, hormone receptor; TN, triple negative.

### Heterogeneity of Costs

Table 4 shows the predictors retained in the final generalized linear models for total per patient costs and costs per patient month. Consistently for both, death in- and outside hospital and an HR/HER2 receptor status other than HR+/HER2- were associated with higher costs, and patient age at diagnosis with lower costs. In addition, systemic and locoregional aggressive therapy were retained as predictors of higher costs and cerebral comorbidity was retained as a predictor of lower costs in the model to explain total per patient costs, but not in the costs per patient month. Moreover, survival time showed a negative association with costs per patient month (mean coefficient estimate = -0.23), but a positive association with total costs per patient (= 0.31). A sensitivity analysis only including the subsample of deceased patients yielded comparable results (ie, most regression coefficients only changed marginally). For the total cost per patient model, the Bayesian information criterion value was 13 626.7, and the McFadden pseudo *R*^2^ was 0.47, indicating an excellent model fit. For the cost per patient month model, the Bayesian information criterion value was 10 327.3, and the pseudo *R*^2^ was 0.40, suggesting a lower but still good fit to the data.

**Table 4:** Factors associated with observed (ie, unadjusted) costs per patient month and costs per patient: results of the generalized linear model with a gamma distribution and a log link function.

	Costs per patient month		Total costs per patient	
	Coefficient∗ (95% CI†)	*P*	Coefficient∗ (95% CI†)	*P*
Intercept	9.14 (8.67-9.61)	<0.001	9.80 (9.36-10.25)	<0.001
Age (years)	-0.02 (-0.03 to -0.02)	<0.001	-0.02 (-0.03 to -0.02)	<0.001
Survival time (years)	-0.23 (-0.28 to -0.18)	<0.001	0.31 (0.27-0.36)	<0.001
Event				
Censored	Reference		Reference	
Death in hospital	0.86 (0.59-1.14)	<0.001	0.72 (0.51-0.93)	<0.001
Death outside hospital	0.18 (-0.03 to 0.39)	0.086	0.38 (0.21-0.54)	<0.001
Cerebral comorbidity			-0.23 (-0.50 to 0.06)	0.091
Any systemic therapy			1.06 (0.83-1.27)	<0.001
Locoregional aggressive treatment			0.23 (0.01-0.46)	0.053
Initial receptor status				
HR+/HER2-	Reference		Reference	
HR+/HER2+	0.51 (0.27-0.75)	<0.001	0.60 (0.41-0.80)	<0.001
HR-/HER2+	0.58 (0.31-0.87)	<0.001	0.66 (0.43-0.90)	<0.001
TN	0.30 (0.07-0.54)	0.016	0.29 (0.09-0.50)	0.005

CI indicates confidence interval; HER2, human epidermal growth factor receptor 2; HR, hormone receptor; TN, triple negative.

∗ The reported coefficients represent the log of the expected change in costs for a unit change in the independent variable. Since effects are multiplicative, regression coefficients cannot be interpreted independently. For example, the effect of 2 years instead of 1 year survival on total costs in a 65-year-old HR+/HER2+ patient with ABC can be estimated using the following formula: exp(9.80+0.31∗2−0.02∗65+0.60)−exp(9.80+0.31∗1−0.02∗65+0.60)=€4437.37.

† Bootstrapped confidence intervals using 1000 iterations.

## Discussion

Our study provides an overview of the real-world costs of patients with ABC in The Netherlands. After adjusting for censoring, the average (SE) lifetime hospital costs of ABC were estimated to be €52 709 (405). However, there was large variation in these costs between the HR/HER2 subtypes, ranging from €29 803 (1130) for patients with TN ABC to €92 272 (910) for patients with HR+/HER2+ ABC. Our analyses further revealed that, underlying this variation, there were considerable differences in the structures of costs. In all groups, medicines, and more specifically only a few expensive agents, accounted for a large share of total costs. In addition to the HR/HER2 receptor status, several other patient characteristics were independently associated with the costs per patients and/or per patient month, including age, survival time, locoregional aggressive treatment, death in hospital, and cerebral comorbidity.

To our knowledge, this is the first study to investigate the real-world lifetime costs of ABC in a cohort, including patients with any HER2/HR receptor status as well as de novo and recurrent ABC, in The Netherlands. A previous real-world study by Frederix et al^23^ included only 88 patients with ABC HER2+, treated in 3 hospitals in The Netherlands between 2004 and 2010. They estimated the lifetime costs to be €48 996. Even though the data collection and analysis methods were similar to ours, the reported resource use and cost estimates were much lower than what we found for both patients with the HR+/HER2+ (€92 272) and the HR-/HER2+ subtype (€69 079). Apart from the shorter follow-up time of 2 years for each patient in the study by Frederix et al, the increasing availability of targeted therapies in recent years should be considered a main reason for the apparent difference in ABC lifetime cost estimates.^23^ Van Kampen et al^24^ also used resource use data from the SONABRE Registry to compare real-world and trial-based cost-effectiveness estimates of bevacizumab in HER2-negative patients with ABC. Their mean lifetime cost estimates range €69 282, for patients receiving taxane monotherapy, and €125 496, for patients receiving bevacizumab. Both estimates are considerably higher than what we found in our study for subgroups of patients with HER2-negative ABC (HR+/HER2- = €47 495; TN = €29 803). It should be noted, however, that the sample in the study of van Kampen et al consists of a selected and small subgroup of patients: only 33 patients who received bevacizumab and 29 controls with HER2-negative ABC were considered. To estimate lifetime costs, observed costs were combined with survival time estimates from a previous study, conducted in a different setting (Canada in 1990s and early 2000s) in a Markov model.^25^^,^^26^

For studies conducted in other settings, similar discrepancies can be observed but are less surprising^4^^,^^11^: For example, a study by Bonastre et al^12^ in a single hospital in France, including 290 patients with ABC, who died between 2005 and 2008, reported ABC mean lifetime costs of €36 516. In contrast, a study in 53 patients with ABC, treated in the Uppsala region in Sweden, who died between 2005 and 2006 incurred hospital lifetime costs of €93 700.^27^ Differences in the organization of health service delivery and in drug reimbursement prices between countries are likely to contribute to the apparent differences in costs.^4^ Nevertheless, across settings, inpatient days and costs for medicines, in particular for trastuzumab, which is specifically highlighted in all 3 aforementioned studies, are found to be main drivers of ABC lifetime costs.^12^^,^^23^^,^^27^

The patient cohort included in our study is somewhat different from other epidemiological cohorts, such as the French ESME^28^: half of the metastases in the ESME population were found asymptomatically (by screening), whereas screening for distant metastases is not standard practice in The Netherlands. Furthermore, the ESME cohort excludes patients not systemically treated, whereas all newly diagnosed patients, including the 11% of patients who did not receive any systematic treatment, were included in SONABRE. In addition, ESME includes comprehensive cancer centers, whereas a mixture of hospital types are participating in SONABRE. These differences could all explain the lower median overall survival observed in our SONABRE population (ie, 24 months) when compared to, for example, the ESME cohort (ie, 37 months).

In contrast to previous cost of illness studies, which either focused on a selective ABC population (eg, including only patients with HER2 positive^23^ or negative^24^ ABC) or had only a small sample size,^11^^,^^27^ we provide precise cost estimates for a general cohort of patients with ABC in The Netherlands, as well as for the most relevant subgroups. Continued consistent data collection by the SONABRE Registry will ensure that our findings can be updated and refined in the future.

There are also several limitations to our study that deserve mention. We investigated costs of ABC from the hospital perspective and collected data in 5 hospitals. Resources that were used in other healthcare sectors (eg, primary care, nursing care, hospice) or in other areas of society (eg, education, judiciary, productivity losses) were not taken into consideration. Hospital resource use was also only collected in 5 hospitals in the southeast of The Netherlands, and even though we do not have any reason to expect major systematic differences, those may not be representative of the country as a whole. Moreover, we did not account for vial wastage, and due to the retrospective data collection, some resource uses may have been missed, which may have led to an underestimation of costs. However, because of the financial implications for the hospitals, the amount of not recorded resource consumption is probably low. For some types of resources, especially for surgical interventions, reference prices^17^ were not available, and it was not always possible to retrieve unit costs from other publicly available sources. In several instances, we had to use internal cost prices from the Maastricht University Medical Centre+, which are confidential and cannot be reported. In other instances, we had to impute missing resource use information, using the hot deck imputation method. Even though the amount of missing data was relatively small, and it is unlikely that it introduced relevant bias into the analysis, it should be noted that the method has important limitations. First, we did not account for important patient characteristics and may have thus generated implausible resource use patterns (eg, use of mutually exclusive treatments). Second, hot deck imputation may lead to an underestimation of the uncertainty around mean estimates. In addition, there are several methods available to estimate lifetime costs in the presence of censoring. Lin’s method has shown to provide accurate results,^20^ but up until now, it is not clear whether a different method would have been more precise. Since some of our analyses were conducted using the observed (ie, unadjusted) patient costs instead of adjusted lifetime costs, results might have also been affected by the censoring of patient time and costs. To account for this in the multivariable regression model, we included death, inside or outside of the hospital, as an explanatory factor, but in other analyses, resource use and costs were probably underestimated. The reported costs thus represent a conservative estimate and should be interpreted as a lower bound. Finally, it should be noted that reported resource use and cost estimates may be context specific. Treatment patterns can be expected to differ between countries and change over time. During the study period, modern, expensive targeted agents, such as CDK4/6 inhibitors in HR+/HER2- ABC, pertuzumab and T-DM1 in HER2+ ABC, and atezolizumab in TN ABC, were available only to a small proportion of patients or were not available at all. The increasing use of these treatments may have already affected the management and the lifetime costs of patients with ABC in The Netherlands, and it can be expected that this trend will continue with the advent of new therapies in the next few years.

Our study provides essential information about the real-world hospital costs of ABC that provides insight into the complex structure of costs in the heterogenous population of patients with ABC. We report several complementary measures of costs, facilitating a comparison of adjusted and unadjusted lifetime costs and monthly costs per patient, which can be used by others in health economic evaluations and to inform health policy. For comparisons with current or past costs, cost estimates can be adjusted using the respective consumer price index.^18^ We also identified several factors that were independently associated with the total hospital costs per patient: in addition to HR/HER2 receptor status, this includes age at diagnosis, survival time, death inside or outside the hospital, any systemic and locoregional aggressive treatment, and the presence of a cerebral comorbidity. When combined with effectiveness data and put into context, the reported estimates can help to improve the quality of decision analytical models and enable more precise subgroup analysis in patients with ABC, which, ultimately, can help inform sound decision making. More research is required to better understand if, and if so, which factors are predictive of healthcare spending in patients with ABC. Future studies should aim to also take into account the longitudinal structure of healthcare costs over time and investigate its temporal dynamics. Furthermore, real-world cost data from outside the health sector are required to complement the information reported in this study.

## Conclusion

We investigated the real-world hospital cost of patients with ABC in The Netherlands. The comprehensive description of resource use and associated costs provides researchers and policy makers with key figures for economic evaluations and budget impact analyses. Our analyses offer new insights into the structure and clearly shows the large heterogeneity of hospital costs of patients with ABC in The Netherlands. A better understanding of the real-world costs of ABC will be increasingly important to inform priority setting and resource allocation in healthcare, as novel and expensive therapies become available.
