# Supplementary material for: Direct Medical Costs of Advanced Breast Cancer Treatment: A Real-World Study in the Southeast of The Netherlands
Source: Value Health. 2021 May;24(5):668–75. doi: 10.1016/j.jval.2020.12.007 (PMC8105643; doi:10.1016/j.jval.2020.12.007)
Supplement: Supplementary Material [file mmc1.docx]

Supplementary data for:

**Direct medical costs of advanced breast cancer treatment: a Real-world Study in the South East of the Netherlands**

- Table S1 shows the unit prices that were used in the study.
- Tables S2–S5 provide HR/HER2 subgroups specific resource use and cost figures for all resource categories, and for three individual resources with the highest share in costs.

| **Table S1: Unit costs (in €2017)** | | | | | | | | | | | | |  |
| --- | --- | --- | --- | --- | --- | --- | --- | --- | --- | --- | --- | --- | --- |
| **Resource** | | **Unit cost** | | **Ref** | | **Resource** | | **Unit cost** | | **Ref** | | |  |
| 5-fluorouracil | | 0.0067 | | 3 | | Docetaxel | | 5.04 | | 3 | | |  |
| Abdominal surgery | | Confidential | | 2 | | Doxorubicin | | 22.02 | | 3 | | |  |
| Ablatio with reconstruction | | Confidential | | 2 | | Drainage of ureter | | Confidential | | 2 | | |  |
| Alendronic acid | | 0.01 | | 3 | | Drug administration | | 130.00 | | 4 | | |  |
| Anastrozol | | 0.03 | | 3 | | Echocardiography | | 78.78 | | 1 | | |  |
| Ascites drainage | | 143.24 | | 1 | | Emergency Dep. visit | | 263.40 | | 1 | | |  |
| Axillary node dissection | | Confidential | | 2 | | Epirubicine | | 6.15 | | 3 | | |  |
| Bevacizumab | | 3.37 | | 3 | | Eribulin | | 480.19 | | 3 | | |  |
| Biopsy | | Confidential | | 2 | | Erythrocytes transfusion | | 219.67 | | 1 | | |  |
| Bone scan | | Confidential | | 2 | | ER-test | | 47.40 | | 5 | | |  |
| Brain/neurosurgery | | Confidential | | 2 | | Etoposide | | 0.17 | | 3 | | |  |
| Brivanib | | 1.00 | | 3 | | Everolimus | | 14.58 | | 3 | | |  |
| CA 15.3 | | Confidential | | 2 | | Exemestane | | 0.0092 | | 3 | | |  |
| Capecitabin | | 0.0027 | | 3 | | Fulvestrant | | 1.30 | | 3 | | |  |
| Carboplatin | | 0.31 | | 3 | | Gemcitabin | | 0.09 | | 3 | | |  |
| Central venous catheter | | Confidential | | 2 | | Gosereline | | 340.58 | | 3 | | |  |
| Clodronic acid | | 0.0041 | | 3 | | HER2 FISH test | | 148.13 | | 5 | | |  |
| CT abdomen | | 192.35 | | 1 | | HER2 IHC | | 47.40 | | 5 | | |  |
| CT other | | 131.98 | | 1 | | Hickmann | | Confidential | | 2 | | |  |
| CT thorax | | 185.19 | | 1 | | Ibandronic acid | | 0.11 | | 3 | | |  |
| CT thorax abdomen | | 377.54 | | 1 | | ICU in-patient day | | 1,206.16 | | 1 | | |  |
| Cyclophosphamide | | 0.03 | | 3 | | In-patient day | | 646.81 | | 1 | | |  |
| Cytarabine | | 1.33 | | 3 | | Lapatinib | | 0.07 | | 3 | | |  |
| Denosumab | | 3.58 | | 3 | | Letrozole | | 0.02 | | 3 | | |  |
| DEXA Bone Densitometry | | 78.78 | | 1 | | Liver surgery | | Confidential | | 2 | | |  |
| **Table S1 (continued): Unit costs (in 2017 €)** | | | | | | | | | | | | | |
| **Resource** | | | **Unit cost** | | **Ref** | | **Resource** | | **Unit cost** | | **Ref** | | |
| Lumpectomy | | | Confidential | | 2 | | Port a cath | | Confidential | | 2 | | |
| Mamma sono | | | 89.01 | | 1 | | PR-test | | 47.40 | | 5 | | |
| Mastectomy | | | Confidential | | 2 | | Pulmonary surgery | | Confidential | | 2 | | |
| Medroxyprogesteron | | | 0.0051 | | 3 | | Radiotherapy | | 252.81 | | 6 | | |
| Megestrol | | | 0.02 | | 3 | | Resection metastasis (other) | | Confidential | | 2 | | |
| Methotrexate | | | 0.23 | | 3 | | Resection of skin metastasis | | Confidential | | 2 | | |
| Mitomycin | | | 3.86 | | 3 | | Risedronic acid | | 0.02 | | 3 | | |
| MRI | | | 257.83 | | 1 | | Secondary reconstruction | | Confidential | | 2 | | |
| MUGA scan | | | 78.78 | | 1 | | Sentinel node procedure | | Confidential | | 2 | | |
| Olaparib | | | 0.24 | | 3 | | Stent bile duct | | Confidential | | 2 | | |
| Orthopedic surgery: extremities | | | Confidential | | 2 | | Stent gastro-intestinal tract | | Confidential | | 2 | | |
| Orthopedic surgery: spine | | | Confidential | | 2 | | Surgery contralateral axilla | | Confidential | | 2 | | |
| Paclitaxel | | | 2.49 | | 3 | | Surgery of nodes (outside) | | Confidential | | 2 | | |
| Palbociclib | | | 1.64 | | 3 | | T-DM1 | | 18.66 | | 3 | | |
| Pamidronic acid | | | 2.02 | | 3 | | Tamoxifen | | 0.01 | | 3 | | |
| Pertuzumab | | | 0.17 | | 3 | | Telephone consultation | | 17.29 | | 1 | | |
| PET and MRI Scan | | | Confidential | | 2 | | Thrombocytes transfusion | | 530.87 | | 1 | | |
| PET low dose | | | Confidential | | 2 | | Trastuzumab | | 4.02 | | 3 | | |
| PET normal | | | Confidential | | 2 | | Vinorelbin | | 2.45 | | 3 | | |
| PICC catheter | | | Confidential | | 2 | | X-ray | | Confidential | | 2 | | |
| Pleura drainage | | | 143.24 | | 1 | | Zoledronic acid | | 34.98 | | 3 | | |
| Policlinic visit | | | 134.24 | | 1 | |  | |  | |  | | |
| 1 | | Ref:  Institute for Medical Technology Assessment. BIJLAGE 1 Kostenhandleiding: Methodologie van kostenonderzoek en referentieprijzen voor economische evaluaties in de gezondheidszorg. Erasmus Universiteit Rotterdam. 2016. | | | | | | | | | | |  |
| 2 | | Maastricht University Medical Centre+. Internal cost prices. 2018. Confidential | | | | | | | | | | |  |
| 3 | | Zorginstituut Nederland. Medicijnkosten. 2018. Available at: https://www.medicijnkosten.nl/ | | | | | | | | | | |  |
| 4 | | Franken MF, Kanters T, Uyl-de Groot C. Insights into healthcare and societal costs of subcutaneous injection and intravenous infusion of trastuzumab for HER2 positive breast cancer and rituximab for non-Hodgkin’s lymphoma in The Netherlands. 2017. Available at: https://www.imta.nl/assets/uploads/2017/12/Abstract-IMTA_2017_1205.pdf | | | | | | | | | | |  |
| 5 | | Essers BA, Seferina SC, Tjan‐Heijnen VC, Severens JL, Novák A, Pompen M, Oron UH, Joore MA. Transferability of model‐based economic evaluations: the case of trastuzumab for the adjuvant treatment of HER2‐positive early breast cancer in the Netherlands. Value in health. 2010 Jun 1;13(4):375-80. doi: 10.1111/j.1524-4733.2009.00683.x | | | | | | | | | | |  |
| 6 | | Timmer-Bonte, JN, Adang, EM, Smit, HJ et al. Cost-effectiveness of adding granulocyte colony-stimulating factor to primary prophylaxis with antibiotics in small-cell lung cancer. J Clin Oncol. 2006; 24: 2991–2997 | | | | | | | | | | |  |

| Table S2: Resource use and associated costs in € – **SUBGROUP: HR+/HER2-** | | | | | | | |  |  |  |  |
| --- | --- | --- | --- | --- | --- | --- | --- | --- | --- | --- | --- |
| Resource | % of total costs within subgroup | | % of costs  within category | Uncond. average costs per patient | Used by patients |  | Cond. average^a^ Units consumed (standard dose^b^) | | Cond. average^a^  costs per patient  (95%CI^c^) | |  |
| Targeted therapy (any) | 28.7 % | |  | 11,163 | 161 (38.7%) |  |  | | | 28,844 (25,290; 32,381) | |
| Everolimus | 15.5 % | | 54.0 % | 6,034 | 114 (27.4%) |  | 151.00 ( 10mg) | | | 22,017 (18,607; 25,723) | |
| Bevacizumab | 11.6 % | | 40.4 % | 4,510 | 60 (14.42%) |  | 12.38 ( 750mg) | | | 31,271 (25,736; 37,474) | |
| Palbociclib | 1.3 % | | 4.4 % | 489 | 14 (3.37%) |  | 71.11 ( 125mg) | | | 14,538 (10,981; 19,201) | |
| Chemotherapy (any) | 9.6 % | |  | 3,752 | 190 (45.67%) |  |  | | | 8,214 (7,200; 9,275) | |
| Doxorubicin | 3.9 % | | 40.7 % | 1,529 | 65 (15.62%) |  | 4.44 ( 100mg) | | | 9,784 (8,761; 10,809) | |
| Paclitaxel | 2.7 % | | 28.5 % | 1,069 | 84 (20.19%) |  | 16.39 ( 130mg) | | | 5,296 (4,655; 5,984) | |
| Eribulin | 1.1 % | | 11.1 % | 417 | 18 (4.33%) |  | 10.03 ( 2mg) | | | 9,630 (7,002; 12,500) | |
| Hormonal therapy (any) | 4.8 % | |  | 1,887 | 357 (85.82%) |  |  | | | 2,199 (1,797; 2,606) | |
| Fulvestrant | 4.6 % | | 95.3 % | 1,798 | 146 (35.1%) |  | 7.88 (500mg) | | | 5,123 (4,328; 5,931) | |
| Bisphosphonates (any) | 8.0 % | |  | 3,116 | 249 (59.86%) |  |  | | | 5,206 (4,756; 5,674) | |
| Denosumab | 2.8 % | | 34.4 % | 1,073 | 61 (14.66%) |  | 17.04 ( 120mg) | | | 7,320 (6,424; 8,319) | |
| Pamidronic acid | 2.5 % | | 31.3 % | 975 | 108 (25.96%) |  | 20.64 ( 90mg) | | | 3,756 (3,153; 4,389) | |
| Clodronic acid | 2.4 % | | 29.6 % | 923 | 106 (25.48%) |  | 558.25 (1600mg) | | | 3,623 (3,251; 4,051) | |
| Transfusions | 0.6 % | |  | 245 | 86 (20.67%) |  |  | | | 1,185 (941; 1,456) | |
| Systemic therapy administration | | 0.4 % |  | 143 | 77 (18.51%) |  |  | | | 775 (677; 873) | |
| Consultations (any) | 32.4 % | |  | 12,613 | 411 (98.8%) |  |  | | | 12,766 (11,748; 13,873) | |
| In-patient day | 20.4 % | | 63.0 % | 7,950 | 309 (74.28%) |  | 16.55 | | | 10,703 (9,735; 11,724) | |
| Policlinic visit | 9.0 % | | 27.8 % | 3,501 | 407 (97.84%) |  | 26.65 | | | 3,578 (3,363; 3,812) | |
| ICU in-patient day | 1.5 % | | 4.7 % | 589 | 19 (4.57%) |  | 10.70 | | | 12,903 (7,576; 19,178) | |
| Emergency Dep. visit | 1.1 % | | 3.3 % | 413 | 283 (68.03%) |  | 2.30 | | | 607 (558; 658) | |
| Diagnostics (any) | 10.2 % | |  | 3,957 | 410 (98.56%) |  | 0.00 | | | 4,015 (3,776; 4,243) | |
| CT thorax abdomen | 3.3 % | | 32.1 % | 1,271 | 318 (76.44%) |  | 4.40 | | | 1,662 (1,527; 1,796) | |
| CA 15.3 | 1.8 % | | 17.8 % | 706 | 384 (92.31%) |  | 14.79 | | | 765 (717; 822) | |
| Radiotherapy | 3.6 % | |  | 1,422 | 210 (50.48%) |  |  | | | 2,817 (2,334; 3,322) | |
| Surgery (any) | 1.7 % | |  | 664 | 126 (30.29%) |  |  | | | 2,192 (1,805; 2,621) | |
| ^a^The conditional average per patient indicates the average resource use or costs conditional on having used the particular resource. ^b^For drugs, the average use per patient represents the average number of times the reported standard dose was administered, conditional on receiving the drug at least once. ^c^95% confidence interval, based on 1,000 bootstrap iterations. | | | | | | | | | | | |

| Table S3: Resource use and associated costs in € – **SUBGROUP: TN** | | | | | | | |  |  |  |  |
| --- | --- | --- | --- | --- | --- | --- | --- | --- | --- | --- | --- |
| Resource | % of total costs within subgroup | | % of costs  within category | Uncond. average costs per patient | Used by patients |  | Cond. average^a^ Units consumed (standard dose^b^) | | Cond. average^a^  costs per patient  (95%CI^c^) | |  |
| Targeted therapy (any) | 26.1 % | |  | 8,400 | 19 (26.76%) |  |  | | | 31,389 (24,471; 38,612) | |
| Bevacizumab | 25.8 % | | 98.7 % | 8,288 | 18 (25.35%) |  | 16.18 (600mg) | | | 32,692 (25,826; 39,440) | |
| Chemotherapy (any) | 15.1 % | |  | 4,860 | 42 (59.15%) |  |  | | | 8,215 (6,145; 10,384) | |
| Paclitaxel | 5.9 % | | 39.1 % | 1,901 | 23 (32.39%) |  | 16.86 (140mg) | | | 5,867 (4,053; 7,961) | |
| Doxorubicin | 4.2 % | | 28.0 % | 1,362 | 11 (15.49%) |  | 3.63 (110mg) | | | 8,788 (6,846; 10,644) | |
| Eribulin | 1.9 % | | 12.6 % | 611 | 7 (9.86%) |  | 5.47 (2.36mg) | | | 6,199 (4,434; 8,576) | |
| Hormonal therapy (any) | 0.0 % | |  | 1 | 4 (5.63%) |  |  | | | 11 (3; 18) | |
| Bisphosphonates (any) | 0.8 % | |  | 252 | 16 (22.54%) |  |  | | | 1,117 (664; 1,546) | |
| Transfusions | 1.1 % | |  | 342 | 13 (18.31%) |  |  | | | 1,870 (1,021; 2,725) | |
| Systemic therapy administration | | 1.0 % |  | 308 | 21 (29.58%) |  |  | | | 1,040 (669; 1,541) | |
| Consultations (any) | 40.1 % | |  | 12,880 | 70 (98.59%) |  |  | | | 13,064 (10,952; 15,186) | |
| In-patient day | 30.5 % | | 76.0 % | 9,793 | 59 (83.1%) |  | 18.22 | | | 11,785 (9,691; 14,022) | |
| Policlinic visit | 7.7 % | | 19.1 % | 2,462 | 68 (95.77%) |  | 19.15 | | | 2,570 (2,152; 3,098) | |
| Emergency Dep. visit | 1.6 % | | 4.0 % | 512 | 61 (85.92%) |  | 2.26 | | | 596 (497; 704) | |
| Diagnostics (any) | 8.6 % | |  | 2,750 | 69 (97.18%) |  |  | | | 2,829 (2,399; 3,237) | |
| CT thorax abdomen | 2.4 % | | 28.2 % | 776 | 46 (64.79%) |  | 3.17 | | | 1,198 (985; 1,444) | |
| MRI | 1.2 % | | 13.9 % | 381 | 40 (56.34%) |  | 2.63 | | | 677 (509; 916) | |
| PET | 1.1 % | | 12.4 % | 341 | 14 (19.72%) |  | 1.14 | | | 1,731 (1,515; 1,947) | |
| Radiotherapy | 5.4 % | |  | 1,734 | 41 (57.75%) |  |  | | | 3,003 (2,071; 4,211) | |
| Surgery (any) | 1.9 % | |  | 609 | 29 (40.85%) |  |  | | | 1,491 (740; 2,405) | |
| ^a^The conditional average per patient indicates the average resource use or costs conditional on having used the particular resource. ^b^For drugs, the average use per patient represents the average number of times the reported standard dose was administered, conditional on receiving the drug at least once. ^c^95% confidence interval, based on 1,000 bootstrap iterations. | | | | | | | | | | | |

| Table S4: Resource use and associated costs in € – **SUBGROUP: HR+/HER2+** | | | | | | | |  |  |  |  |
| --- | --- | --- | --- | --- | --- | --- | --- | --- | --- | --- | --- |
| Resource | % of total costs within subgroup | | % of costs  within category | Uncond. average costs per patient | Used by patients |  | Cond. average^a^ Units consumed (standard dose^b^) | | Cond. average^a^  costs per patient  (95%CI^c^) | |  |
| Targeted therapy (any) | 56.8 % | |  | 46,381 | 47 (72.31%) |  |  | | | 64,144 (51,187; 78,200) | |
| Trastuzumab | 50.8 % | | 89.6 % | 41,541 | 45 (69.23%) |  | 38.31 (390mg) | | | 60,003 (48,262; 72,514) | |
| T-DM1 | 3.4 % | | 5.9 % | 2,755 | 7 (10.77%) |  | 5.96 (230mg) | | | 25,585 (15,935; 36,354) | |
| Everolimus | 1.7 % | | 2.9 % | 1,361 | 5 (7.69%) |  | 121.3 (10mg) | | | 17,687 (4,520; 34,221) | |
| Chemotherapy (any) | 6.3 % | |  | 5,122 | 46 (70.77%) |  |  | | | 7,237 (5,471; 9,272) | |
| Docetaxel | 1.9 % | | 30.2 % | 1,547 | 23 (35.38%) |  | 4.5 (193mg) | | | 4,373 (3,472; 5,412) | |
| Doxorubicin | 1.9 % | | 29.8 % | 1,525 | 9 (13.85%) |  | 5.56 (90mg) | | | 11,014 (6,462; 16,188) | |
| Paclitaxel | 1.3 % | | 21.2 % | 1,088 | 13 (20%) |  | 14.58 (150mg) | | | 5,439 (4,815; 6,041) | |
| Hormonal therapy (any) | 2.4 % | |  | 1,951 | 52 (80%) |  |  | | | 2,439 (1,358; 3,847) | |
| Fulvestrant | 2.3 % | | 95.4 % | 1,861 | 24 (36.92%) |  | 7.76 (500mg) | | | 5,041 (3,077; 7,648) | |
| Bisphosphonates (any) | 4.7 % | |  | 3,818 | 40 (61.54%) |  |  | | | 6,204 (5,085; 7,351) | |
| Pamidronic acid | 2.3 % | | 50.1 % | 1,912 | 25 (38.46%) |  | 27.32 (90mg) | | | 4,970 (4,073; 5,910) | |
| Denosumab | 1.5 % | | 33.0 % | 1,260 | 10 (15.38%) |  | 19.06 (120mg) | | | 8,189 (6,016; 10,781) | |
| Transfusions | 0.3 % | |  | 285 | 13 (20%) |  |  | | | 1,424 (808; 2,203) | |
| Systemic therapy administration | | 0.9 % |  | 774 | 35 (53.85%) |  |  | | | 1,437 (1,081; 1,809) | |
| Consultations (any) | 19.3 % | |  | 15,775 | 65 (100%) |  |  | | | 15,775 (13,145; 18,456) | |
| In-patient day | 12.7 % | | 66.0 % | 10,409 | 51 (78.46%) |  | 20.51 | | | 13,266 (10,717; 15,841) | |
| Policlinic visit | 5.0 % | | 25.8 % | 4,077 | 65 (100%) |  | 30.37 | | | 4,077 (3,519; 4,641) | |
| Diagnostics (any) | 6.5 % | |  | 5,294 | 65 (100%) |  |  | | | 5,294 (4,542; 5,996) | |
| CT thorax abdomen | 2.2 % | | 34.4 % | 1,824 | 56 (86.15%) |  | 5.61 | | | 2,117 (1,699; 2,542) | |
| CA 15.3 | 1.0 % | | 16.0 % | 849 | 63 (96.92%) |  | 16.95 | | | 876 (741; 1,018) | |
| Radiotherapy | 2.0 % | |  | 1,645 | 35 (53.85%) |  |  | | | 3,055 (2,189; 4,219) | |
| Surgery (any) | 0.8 % | |  | 662 | 27 (41.54%) |  |  | | | 1,593 (914; 2,323) | |
| ^a^The conditional average per patient indicates the average resource use or costs conditional on having used the particular resource. ^b^For drugs, the average use per patient represents the average number of times the reported standard dose was administered, conditional on receiving the drug at least once. ^c^95% confidence interval, based on 1,000 bootstrap iterations. | | | | | | | | | | | |

| Table S5: Resource use and associated costs in € – **SUBGROUP: HR-/HER2+** | | | | | | | |  |  |  |  |
| --- | --- | --- | --- | --- | --- | --- | --- | --- | --- | --- | --- |
| Resource | % of total costs within subgroup | | % of costs  within category | Uncond. average costs per patient | Used by patients |  | Cond. average^a^ Units consumed (standard dose^b^) | | Cond. average^a^  costs per patient  (95%CI^c^) | |  |
| Targeted therapy (any) | 53.3 % | |  | 32,573 | 29 (64.44%) |  |  | | | 50,544 (34,007; 69,664) | |
| Trastuzumab | 44.9 % | | 84.2 % | 27,430 | 29 (64.44%) |  | 17.67 (600mg) | | | 42,564 (27,377; 60,753) | |
| T-DM1 | 6.7 % | | 12.5 % | 4,084 | 4 (8.89%) |  | 10.71 (230mg) | | | 45,940 (15,018; 76,863) | |
| Chemotherapy (any) | 7.0 % | |  | 4,268 | 29 (64.44%) |  |  | | | 6,622 (4,642; 8,602) | |
| Doxorubicin | 2.1 % | | 30.7 % | 1,308 | 6 (13.33%) |  | 4.09 (109mg) | | | 9,812 (7,573; 12,322) | |
| Paclitaxel | 1.8 % | | 25.6 % | 1,092 | 11 (24.44%) |  | 17.96 (100mg) | | | 4,466 (3,457; 5,381) | |
| Docetaxel | 1.4 % | | 20.4 % | 869 | 9 (20%) |  | 4.31 (200mg) | | | 4,344 (3,355; 5,203) | |
| Hormonal therapy (any) | 0.0 % | |  | 2 | 2 (4.44%) |  |  | | | 36 (3; 69) | |
| Bisphosphonates (any) | 1.9 % | |  | 1,137 | 11 (24.44%) |  |  | | | 4,652 (2,933; 6,654) | |
| Transfusions | 0.3 % | |  | 163 | 10 (22.22%) |  |  | | | 734 (523; 963) | |
| Systemic therapy administration | | 1.2 % |  | 716 | 21 (46.67%) |  |  | | | 1,535 (1,164; 1,944) | |
| Consultations (any) | 25.3 % | |  | 15,448 | 45 (100%) |  |  | | | 15,448 (12,130; 19,088) | |
| In-patient day | 18.9 % | | 74.9 % | 11,571 | 39 (86.67%) |  | 20.64 | | | 13,351 (10,266; 16,801) | |
| Policlinic visit | 5.1 % | | 20.0 % | 3,094 | 42 (93.33%) |  | 24.69 | | | 3,315 (2,582; 4,127) | |
| Diagnostics (any) | 6.5 % | |  | 3,941 | 45 (100%) |  |  | | | 3,941 (3,049; 4,853) | |
| CT thorax abdomen | 1.5 % | | 23.0 % | 906 | 32 (71.11%) |  | 3.38 | | | 1,274 (991; 1,605) | |
| Radiotherapy | 2.5 % | |  | 1,528 | 21 (46.67%) |  |  | | | 3,275 (2,155; 4,454) | |
| Surgery (any) | 2.0 % | |  | 1,213 | 17 (37.78%) |  |  | | | 3,211 (1,727; 4,635) | |
| ^a^The conditional average per patient indicates the average resource use or costs conditional on having used the particular resource. ^b^For drugs, the average use per patient represents the average number of times the reported standard dose was administered, conditional on receiving the drug at least once. ^c^95% confidence interval, based on 1,000 bootstrap iterations. | | | | | | | | | | | |
